# Supplementary material for: Multi-F-Structured MOF Materials Enhance Nanogenerator Output Performance for Corrosion Protection of Metallic Materials
Source: Molecules. 2023 Dec 1;28(23):7894. doi: 10.3390/molecules28237894 (PMC10707780; doi:10.3390/molecules28237894)
Supplement: Supplementary file 1 [file molecules-28-07894-s001.zip › molecules-2712852-supplementary.pdf]

Supporting Information

Table of Contents

1. Experiments 3

2. Assembly of TENG's working principle 4

3. Characterization 4

4. Graphs 5

1.Experiments

1.1 Materials

The chemicals and reagents involved in this experiment were obtained by purchasing through commercial platforms and were used directly without further purification.

1.2 Synthesis of compounds **1-2**

Synthesis of [Co (2,3,5,6-tetrafluoroterephthalic acid)] (1). A solid mixture of $Co(NO_3)_2$-$6H_2O$ (29.1 mg, 0.1 mmol) and 2,3,5,6-tetrafluoroterephthalic acid (23.8 mg, 0.1 mmol) was weighed and dissolved in a mixture of DMF (2 mL), water (2 mL), and ethanol (6 mL), and the resultant solution was transferred to a small glass vial with a pipette gun, and the vial was opened and allowed to evaporate naturally at room temperature, light purple crystals were precipitated after three Wednesdays, washed three times with ethanol and dried at room temperature.

Synthesis of [Co (2,5-bis(trifluoromethyl)terephthalic acid)] (2). A solid mixture of $Co(NO_3)_2$-$6H_2O$ (15.2 mg, 0.05 mmol) and 2,5-bis(trifluoromethyl)terephthalic acid (15.2 mg, 0.05 mmol) was weighed and dissolved in a mixture of DMF (6 mL), water (2 mL), and ethanol (2 mL), and the resultant solution was transferred to a reactor using a pipette gun, which was placed in a pre The reaction was carried out in an oven heated up to 80 °C for 24 h. The solution was cooled to room temperature, washed three times with DMF and placed in a vacuum drying oven for drying.

1.3 Preparation of 1/2 base TENG

TENG positive electrode sheet preparation: the compound **1** and **2** grinding evenly coated in 5cm × 6cm copper sheet, and will be coated with conductive silver epoxy resin of the copper wire fixed in the other side of the copper sheet, in the fixed copper wire of the copper tape surface covered with a layer of transparent adhesive tape, and finally cut it out into 5cm × 5cm.

TENG negative electrode sheet (PVDF) preparation: weighing the appropriate amount of PVDF powder dissolved in a mixture of DMA and acetone, stirring at 60 °C until the PVDF completely dissolved to stop the heating, cooled to room temperature, through the desktop homogenizer will be uniformly coated with the PVDF mixture on the Kapton membrane, the

spin-coating of the membrane is placed in the oven drying at 80 ℃ for 4 h and then remove the cooled to room temperature. room temperature. A copper tape of 5 cm × 6 cm was applied to the reverse side of the PVDF film, and the negative electrode sheet was prepared in the same way as the positive electrode sheet.

2.Working principle of assembling TENG

Fig. S4i represents the initial state of the potential difference of the external circuit without external force; when a certain external force is applied, the two electrodes are in contact with each other, and an equal number of charges but with opposite signs will be generated in the PVDF and compound layers, and the PVDF layer is negatively charged with respect to the compound electrode layer (ii); when the external force is removed, the potential difference between the upper and lower electrodes will be generated due to electrostatic induction (iii), and the potential difference will disappear when the The potential difference disappears when the charge reaches equilibrium again (iv); when the same external force is applied to the TENG system again, the distance between the two layers of electrode material changes again, which causes the potential difference between the two electrodes to change as well (the potential difference decreases with the decrease of the distance), and thus the electrons flow from the PVDF electrode to the compound layer (Fig. V), until the charge accumulates to reach the new charge equilibrium (ii).

3. Characterization

Thermogravimetric analysis (TGA) data were collected on a Netzsch STA 449C thermal analyzer. Compounds were subjected to Mott - Shottky test by a three-electrode system on an electrochemical workstation (CHI 660E, Shanghai Chenhua Instrument Co., Ltd., China). The chemical bond determination of the compounds was tested by FT-IR; the purity was characterized by powder X-ray diffraction mapping (PXRD) under Cu-kα irradiation using an instrument manufactured by Bruker D8 Advance. For testing the elemental composition and valence states XPS was used to obtain spectral data plots of the compounds using Al-Kα as the ray source. The morphology, elemental composition and distribution of the compounds were tested with a German-made FE-SEM and its accompanying EDS. The Isc and Voc of the friction nanogenerators were tested on an SR570 low-noise current amplifier (Stanford Research Systems) and a 2657A high-power system source meter.The bandgap size of the

samples was tested with a UV-2600 UV-Vis diffuse reflectance spectrometer. The crystal structure was determined by X-ray single-crystal diffractometer, which can solve the structure of the crystals, mainly using the D8 Venture model instrument from Bruker, USA, the main synthesized single crystals were structurally analyzed; the test target was a molybdenum target, the temperature was 298 K, the voltage was 50 KV, and Mo-Kα rays were used, and the solved single-crystal structure was graphically plotted by the Diamond software.

The electrolyte for the Mott - Shottky test was Na_2SO_4 solution at a concentration of 0.2 mol/L. The three-electrode system had the counter electrode connected to a Pt electrode, the reference electrode was an Ag/AgCl electrode, and the working electrode was connected to a conducting glass loaded with compounds **1** and **2**. The tests were carried out with the Impedance-Potential module in the Shanghai Chenhua electrochemical workstation with the voltage test range of -1--1 V and the test frequencies of 500 Hz, 1000 Hz, and 1500 Hz.

The TENG cathodic protection system starts with a 0.3 cm × 0.3 cm square of carbon steel surface appropriately treated for self-powered cathodic protection testing. In the self-powered cathodic protection system, a 3.5 wt% aqueous sodium chloride solution was used to simulate seawater, and the TENG output signal was converted from AC to rectified by connecting a rectifier bridge, the positive pole of which was connected to a platinum electrode, and the negative pole was connected to the carbon steel. The effectiveness of the protection was determined by observing and comparing the surface morphology of the carbon steel with and without the TENG connected.

4. Diagrams

Table S1. Crystallographic data and structure refinement parameters for compounds 1-2

Complex	1	2
empirical formula	$CoC_8H_8F_4O_8$	$CoC_8H_6F_6O_8$
crystal system	monoclinic	monoclinic
space group	$P2_1/n$	$P2_1/c$
a[Å]	5.3295(14)	13.9440(8)
b[Å]	10.246(3)	14.3616(9)
c[Å]	17.239(5)	21.5044(9)
α[°]	90	90
β[°]	94.207(3)	130.423(2)
γ[°]	90	90
V[$Å^3$]	938.82(40)	3278.39(30)
Z	2	4

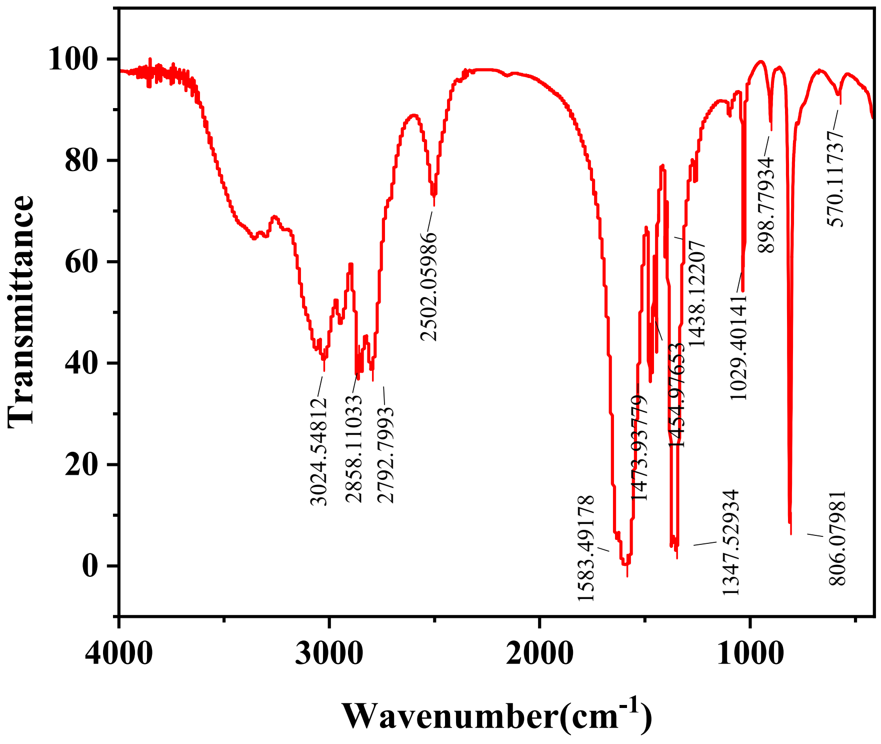

Figure. S1 FT-IR of compound **1**.

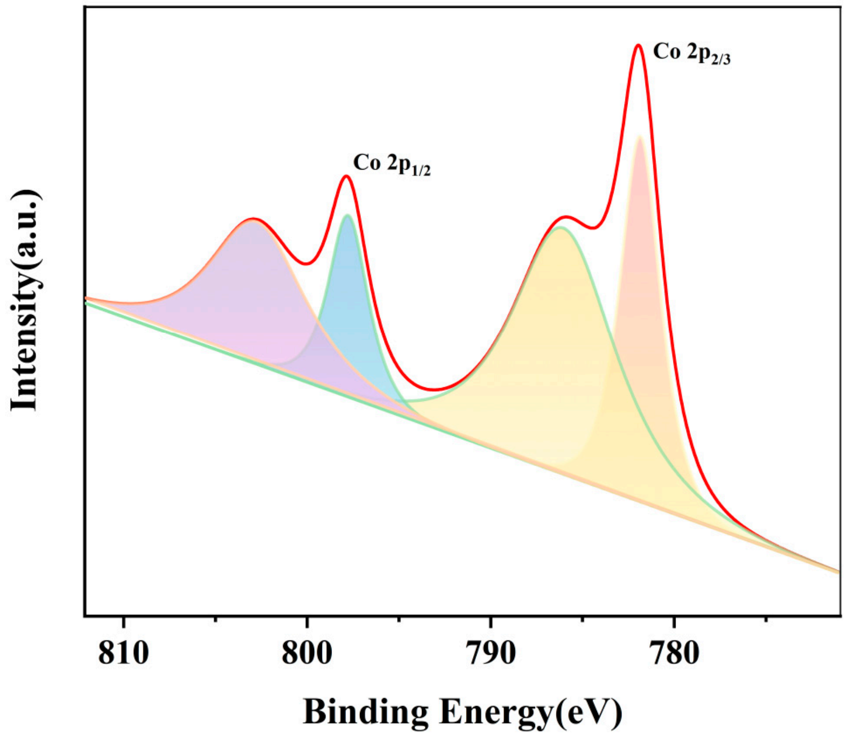

Figure. S2 XPS profile of Co ion in compound **1**

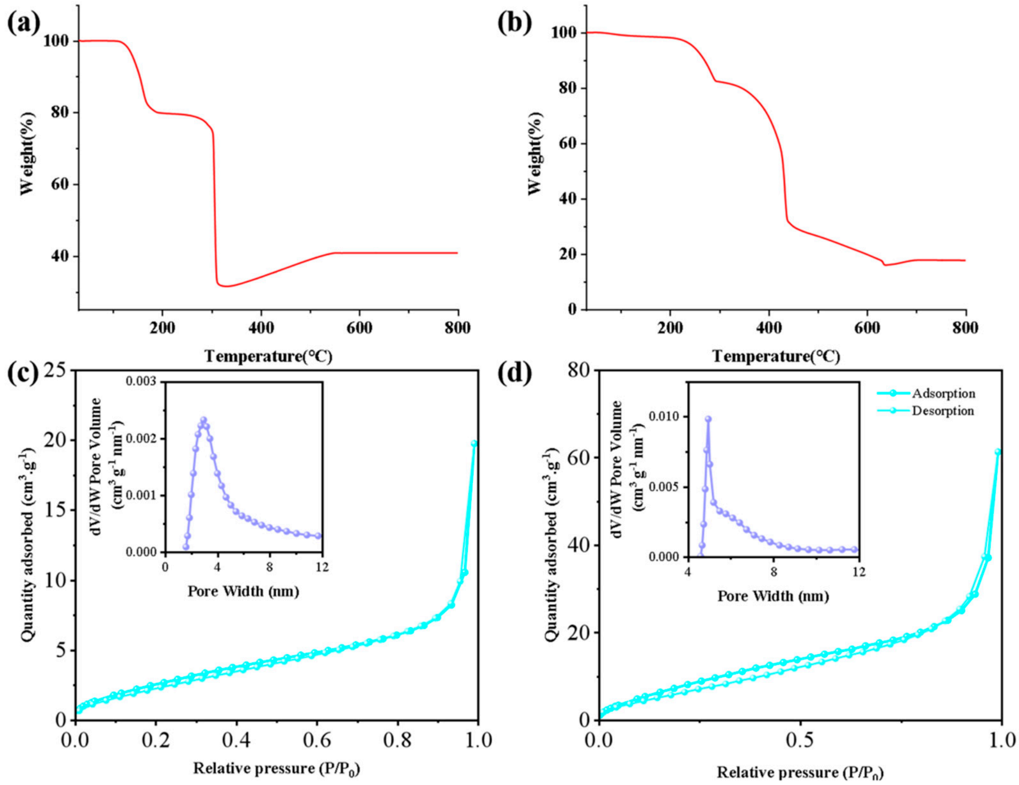

Figure.S3 (a-b) TGA of compounds **1** and **2**; (c-d) N_2 adsorption-desorption isotherms of compounds **1** and **2**.

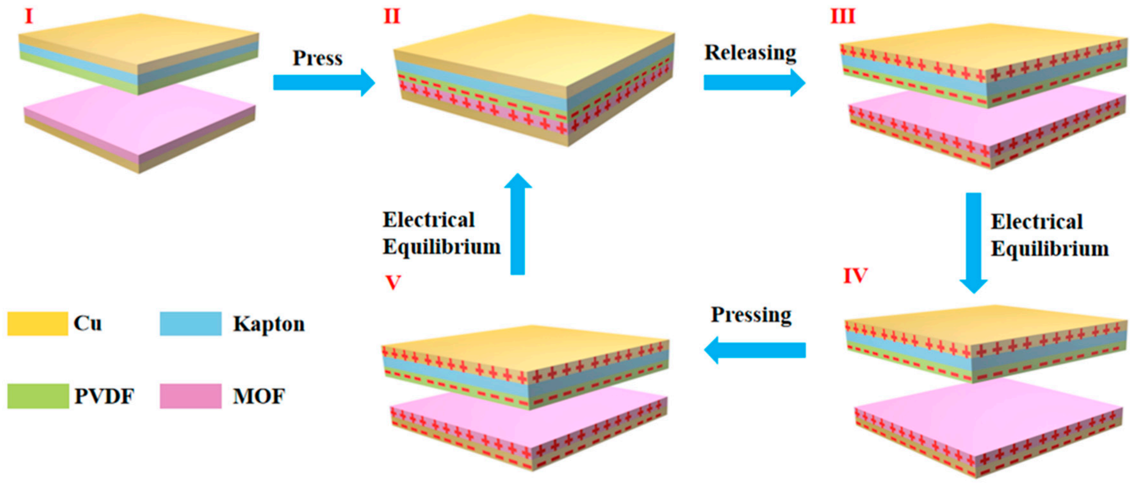

Figure. S4 Mechanism diagram of TENG device operation.

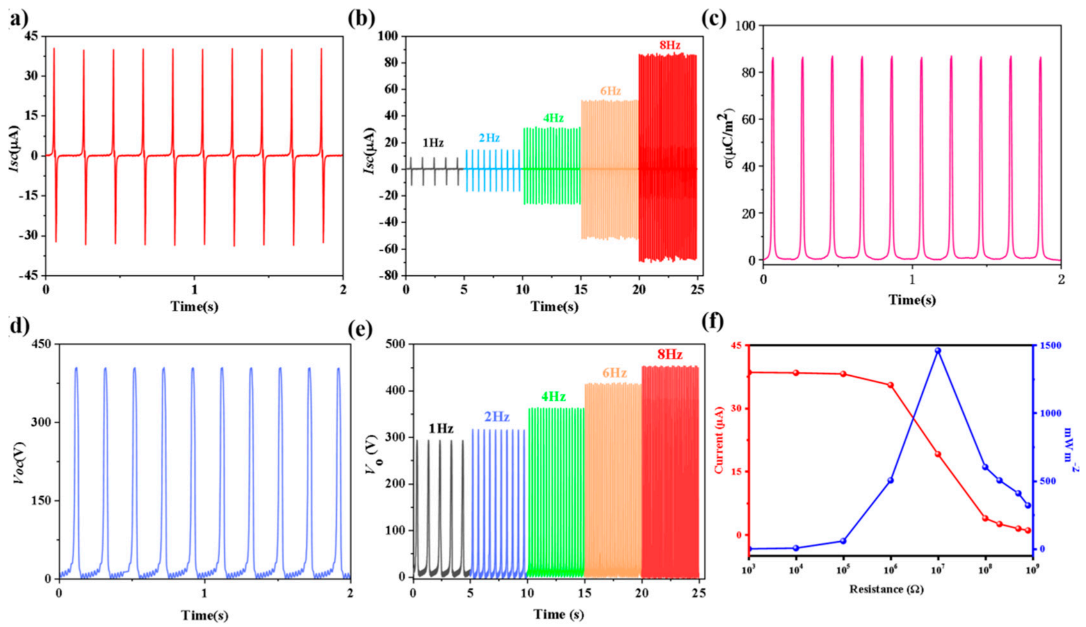

Figure. S5 (a) Isc, (c) σ, (d) Voc, (f) **1**-TENG power density at 5 Hz; (b), (e) Isc vs. Voc for **1**-TENG at different frequencies from 1 to 8 Hz.

A frictional power generator **1**-TENG was made for testing under the same test conditions using **1** as the electrode material. When **1** was operated at 5 Hz, the peak values of short-circuit current and open-circuit voltage were 40.4 μA (Fig. a) and 406.4 V (Fig. d), and the charge density was 87.0 μC m^{-2} (Fig. c). Depending on the power density and short-circuit current of the friction nanogenerator at different load resistances, the instantaneous power reaches a peak of 1455.1 mW m^{-2} when the load resistance is 10 MΩ (Fig. f). As shown in Figs. b and e, the output performance of the TENG, Isc and Voc, increases gradually with increasing frequency, reaching 85.7 μA and 452.0 V at 8 Hz.

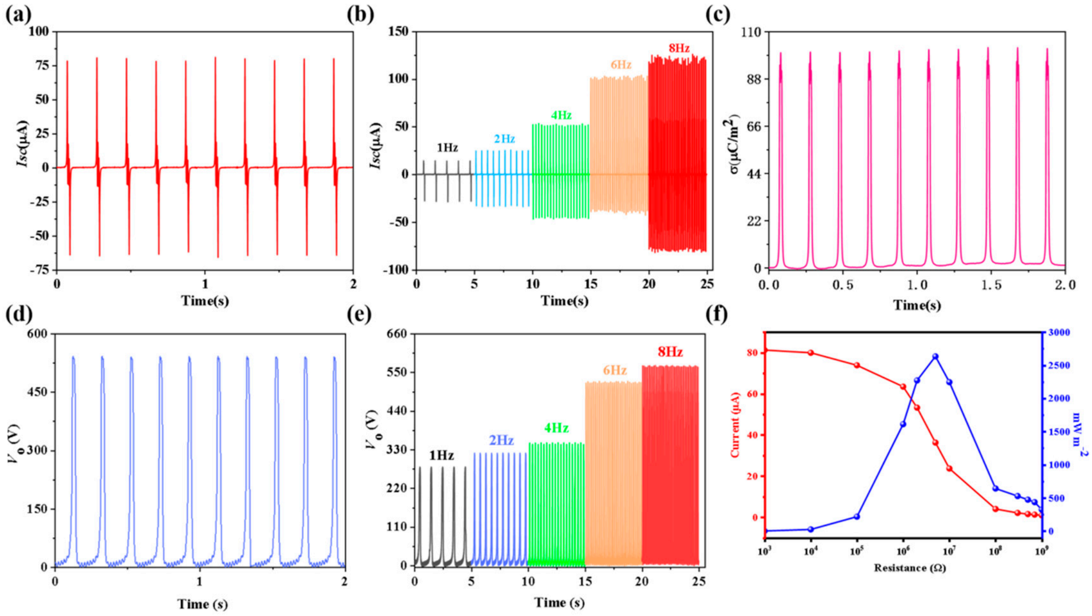

Figure. S6 (a) Isc, (c) σ, (d) Voc, (f) **2**-TENG power density at 5 Hz; (b), (e) Isc vs. Voc for **2**-TENG at different frequencies from 1 to 8 Hz.

A frictional power generator **2**-TENG was made for testing under the same test conditions using **2** as the electrode material. When **2** was operated at 5 Hz, the peak values of short-circuit current and open-circuit voltage were 80.5 μA (Fig. a) and 543.8 V (Fig. d), and the charge density was 101.2 μC m^{-2} (Fig. c). Depending on the power density and short-circuit current of the friction nanogenerator at different load resistances, the instantaneous power reaches a peak of 2629.7 mW m^{-2} when the load resistance is 10 MΩ (Fig. f). As shown in Figs. b and e, the output performance of the TENG, Isc and Voc, increases gradually with increasing frequency, reaching 121.7 μA and 526.0 V at 8 Hz.

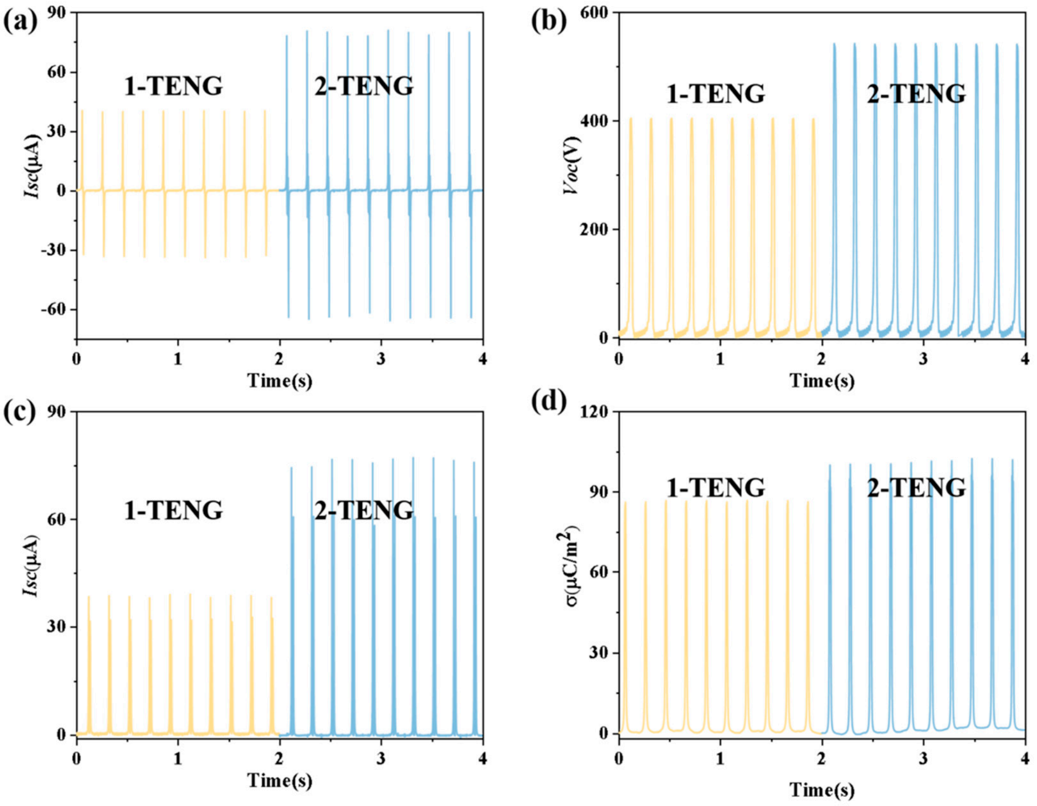

Figure. S7 Comparison plots of **1**-,**2**-TENG at 5 Hz (a) current comparison , (b) voltage comparison, (c) rectification comparison, (d) charge density comparison.

The short-circuit current (Isc) and output voltage (Vo) of **1-**, **2**-TENG at 5 Hz were 40.4 μA and 406.4 V , 80.5 μA and 543.8 V, respectively. The rectifying and charge densities (σ) were 39.0 μA and 87.0 μC m^{-2}, 76.8 μA and 101.2 μC m^{-2}, respectively.

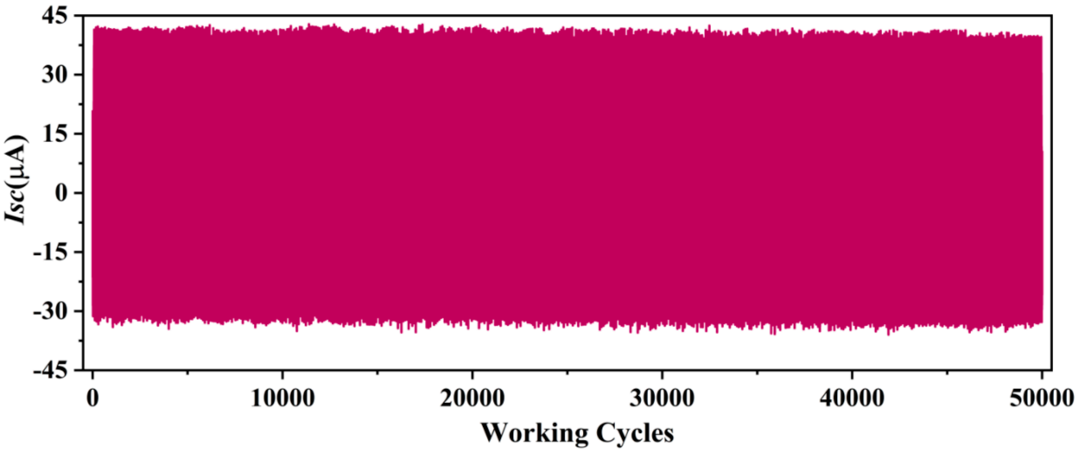

Figure. S8 Isc of **1**-TENG after 50,000 cycles.

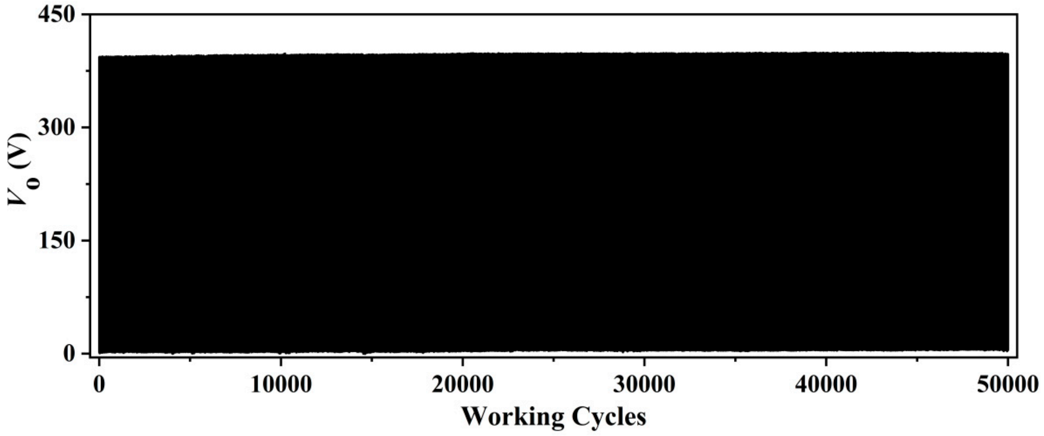

Figure. S9 Voc of **1**-TENG after 50,000 cycles.

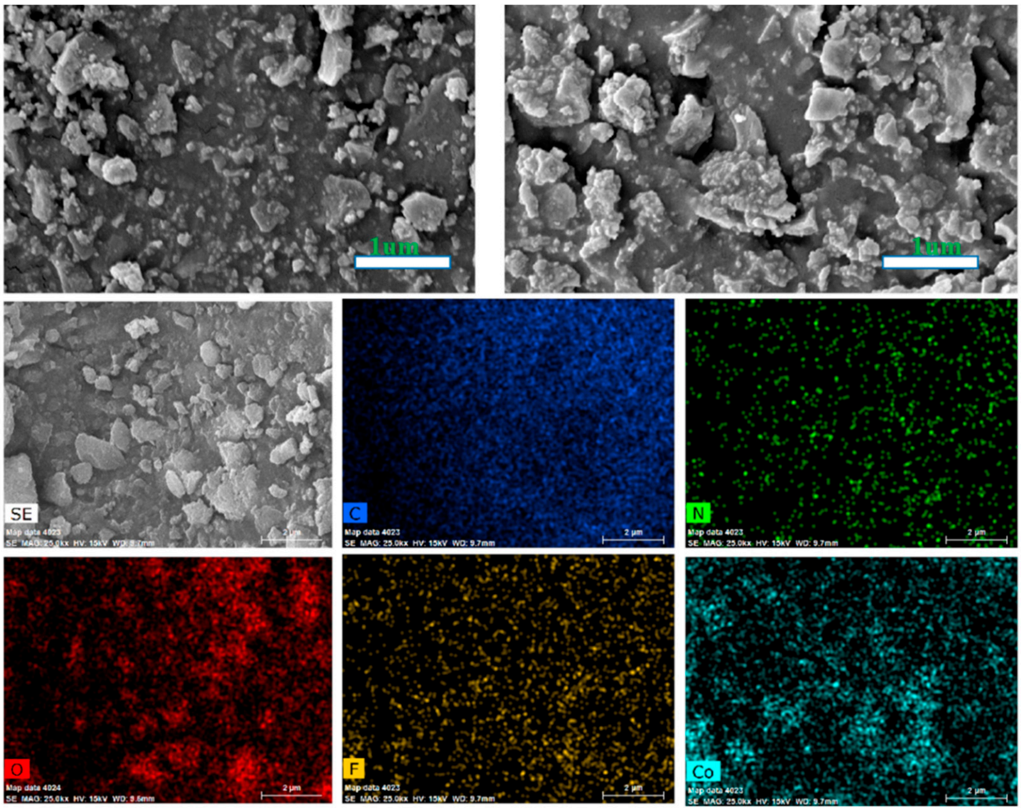

Figure. S10 (a) FE-SEM image of compound **1** before testing; (b) SEM image of compound **1** after testing; (c-h) EDX-mapping analysis of each element in compound **1**.

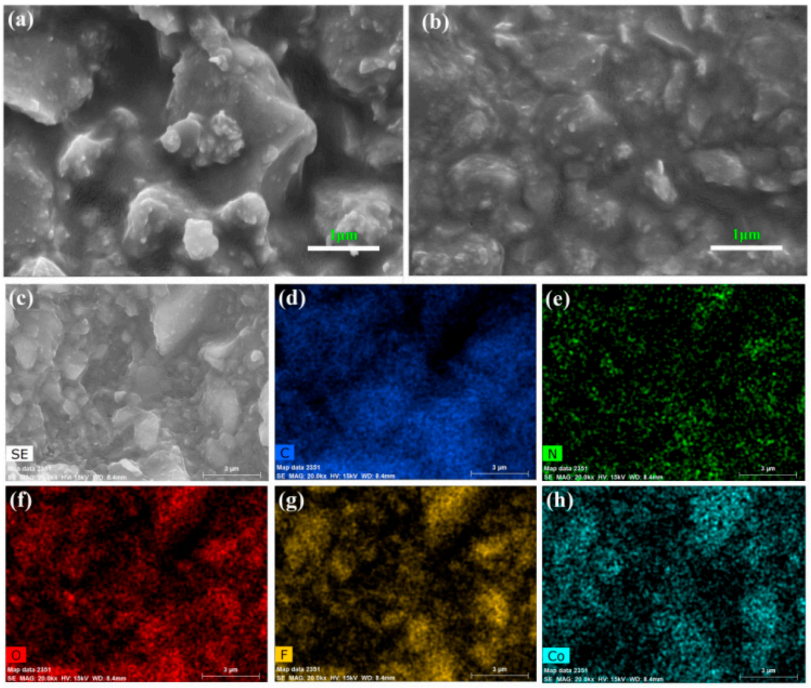

Figure. S11 (a) FE-SEM image of compound **2** before testing; (b) SEM image of compound **2** after testing; (c-h) EDX-mapping analysis of each element in compound **2**.

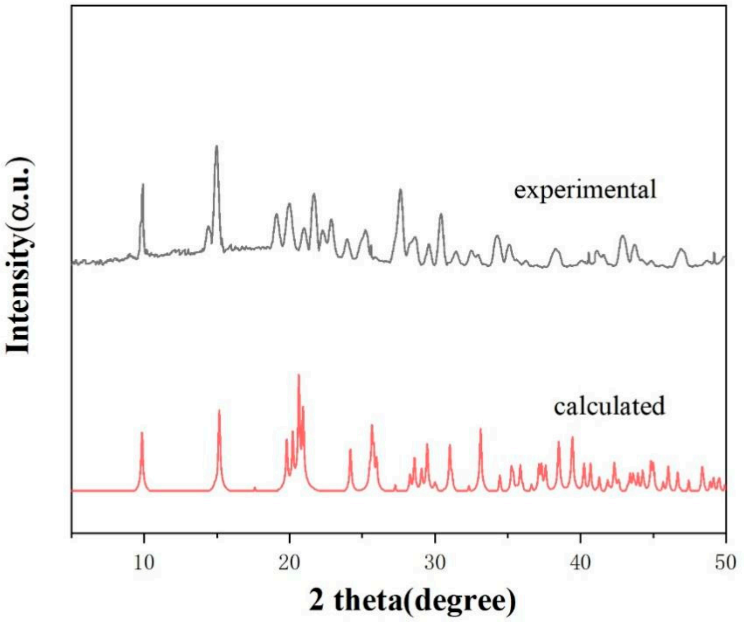

Figure. S12 XRD of compound **1**.
